# Uterine Artery Doppler in Pregnancy: Women with PCOS Compared to Healthy Controls

**DOI:** 10.1155/2018/2604064

**Published:** 2018-08-16

**Authors:** Solhild Stridsklev, Øyvind Salvesen, Kjell Åsmund Salvesen, Sven M. Carlsen, Eszter Vanky

**Affiliations:** ^1^Department of Obstetrics and Gynecology, St. Olav's Hospital, Trondheim University Hospital, Trondheim, Norway; ^2^Department of Clinical and Molecular Medicine, Norwegian University of Science and Technology, Trondheim, Norway; ^3^Department of Public Health and General Practice, Norwegian University of Science and Technology, Trondheim, Norway; ^4^Department of Endocrinology, St. Olav's Hospital, Trondheim University Hospital, Trondheim, Norway

## Abstract

The objective of this study was to investigate possible differences in uterine artery pulsatility index (UtAPI) between pregnant women with PCOS and healthy controls and to explore possible effects of metformin on UtAPI. *Material and Methods*. The study was conducted in a tertiary center. Forty-eight pregnant women diagnosed with PCOS before pregnancy and 124 healthy pregnant women were included. Women with PCOS were randomly assigned to metformin 2000 mg daily or a placebo. UtAPI was measured five times during 1st and 2nd trimesters of pregnancy in women with PCOS and four times in healthy controls. *Results*. There was no difference in UtAPI between PCOS women and healthy controls at any point in time (*p* = 0.34–0.77). In women with PCOS, randomly assigned to metformin 2000 mg or placebo, UtAPI was unaffected by metformin two hours after intake of the first dose of study medication (*p* = 0.34). All PCOS women, regardless of randomization, had higher UtAPI two hours after intake of study medication and a meal compared to before a meal (*p* = 0.02). *Conclusions*. In the first and second trimesters of pregnancy, there was no difference in UtAPI between women with PCOS and healthy controls. Metformin had no immediate effect on the UtAPI. Interestingly, blood flow decreased after a meal, suggesting that time since last meal should be taken into consideration when interpreting the results of UtAPI measurements in pregnancy. This trial is registered with ClinicalTrials.gov (NCT00466622) Metformin in Pregnant PCOS women (PregMet) (NCT00159536).

## 1. Introduction

Over the last twenty years Doppler ultrasound has become a reliable and frequently used method to monitor the fetoplacental unit of risk pregnancies [1–6]. Increased resistance in the uterine artery measured by the pulsatility index indicates a decreased blood flow to the placenta and may be an early sign of placenta pathology and/or hypertensive disorder in pregnancy [1, 6, 7]. PCOS is linked to pregnancy complications, such as gestational diabetes mellitus, preterm delivery, and preeclampsia [8–10]. Studies of UtAPI in women with PCOS are sparse, but some have reported decreased blood flow in the uterine artery in both nonpregnant and pregnant women with PCOS [11–14]. One study reported a significantly higher rate of PCOS women with abnormal UtAPI measurements during first and mid-second trimesters compared to controls [15]. Metformin is an old antidiabetic drug and is known to reduce fasting insulin and testosterone levels in nonobese, nonpregnant women with PCOS [16]. Metformin has also been shown to have a possible vessel-relaxing effect, with increased blood flow in both nonpregnant and pregnant women with PCOS. Results supporting this have been published both before and after we initiated this study [17–19]. We were not able to demonstrate a vessel-relaxing effect in a former study addressing this issue [20]. No studies have accounted for how soon after drug intake this effect can occur.

We hypothesized that unfavourable hemodynamic adaptations in early pregnancy explain why pregnancy complications are more common in women with PCOS. We aimed to study possible differences in UtAPI between women with PCOS and healthy controls and possible immediate effects of metformin on UtAPI.

## 2. Materials and Methods

The present study comprises a subgroup of women with PCOS who participated in the PregMet study, which was an RCT comparing metformin 2 g daily to placebo on the effect of pregnancy complications [21]. Women included in the present substudy underwent extended ultrasound examinations, that is, Doppler of the uterine artery, in addition to following the protocol of the main study [21]. Women were included from February 2005 to September 2008. As controls for this substudy, we recruited healthy women in a prospective observational study. Based on the healthy controls, we constructed a reference curve for UtaPI. The reference curve has been published elsewhere [22]. Healthy control women were included from July 2008 to May 2010. Participants, both women with PCOS and healthy controls, were recruited from general practitioners, the gynecological outpatient clinic of the hospital, and on the basis of “word of mouth.” All women were recruited during first trimester of pregnancy. Biometric variables, including height, weight, and blood pressure were recorded.

The Committee for Medical Research Ethics of Health Region IV, Norway, approved all of the studies ((1) controls 4.2008.841, (2) PregMet 145.04, and (3) the substudy FlowMet 4.2007.97). The substudy was registered separately at http://www.clinicaltrials.gov (NCT00466622) (PregMet NCT00159536). The study on healthy controls was an observational study and not registered in any trial register. Written informed consent was obtained from each patient before inclusion, and the Declaration of Helsinki was followed throughout the studies.

The PregMet study was a multicenter randomized controlled trial (RCT) in which women received metformin or placebo. There was no difference at baseline between the groups during 1st and 2nd trimesters [21]. Inclusion criteria for the PregMet study were (1) PCOS diagnosed according to the Rotterdam criteria [23], (2) age 18–45 years, (3) gestational age between 5 and 12 weeks, and (4) a single viable fetus shown on ultrasonography. The exclusion criteria were alanine aminotransferase (ALAT) > 90 IU/L, serum creatinine concentration > 130 *μ*mol/L, known alcohol abuse, previously diagnosed diabetes mellitus or fasting s-glucose > 7.0 mmol/L at the time of inclusion, treatment with oral glucocorticoids, or use of drugs known to interfere with metformin. PCOS women were followed up during the entire pregnancy and after delivery. A detailed description is published elsewhere [21].

**Figure 1:**
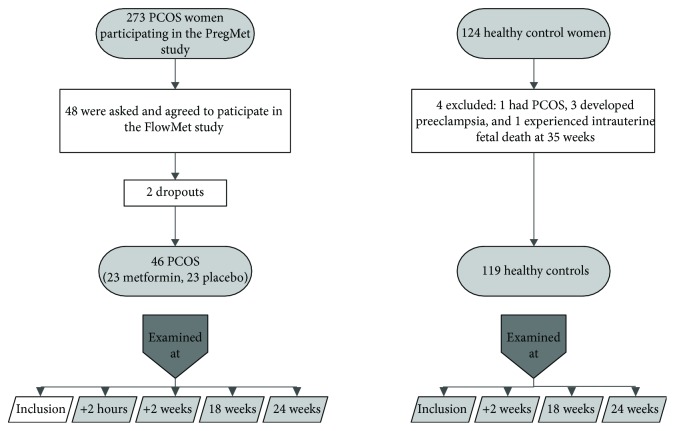
Participants and examinations. “+2 hours” is two hours after the first examination, and “+2 weeks” is 2 weeks after the first examination.

Out of 273 women participating in the PregMet study, 48 were asked and agreed to participate in a substudy called the FlowMet study (Figure 1). Women were asked to participate if they lived close to the hospital, thus making the extra visits feasible. Women were not compensated for participation but were offered a free meal between or after examinations. Women included in the FlowMet study underwent four additional ultrasound examinations during the study period, and otherwise, they followed the PregMet study protocol [21]. These women participating in the substudy were comparable to the whole group of women with PCOS regarding baseline characteristics.

The first UtAPI examination was performed in the morning after an overnight fast. Immediately after the examination, women were instructed to take the first dose of study medication, metformin 500 mg or an oral placebo, and were also given a meal (a sandwich and an optional drink). Two hours after tablet intake, women returned for a second examination. We intended to investigate if metformin had an immediate effect on the uterine arteries and the placental circulation expressed as an altered UtAPI. This second nonfasting measurement was the one used in the analyses comparing PCOS women with controls (as the controls were not fasting). The third examination was performed two weeks after inclusion when the women reached the full dose of study medication (metformin 1000 mg × 2 or placebo). This examination was not done in a fasting state, and we did not record time since last meal or time of day. Examination 4 was done in the morning after an overnight fast at gestational week 18, and examination 5 was done at gestational week 24 in a nonfasting state. Time since last meal or time of day was not recorded.

As controls, we included 124 healthy pregnant women in a prospective observational study. Inclusion criteria in this study were (1) healthy women with an ongoing first trimester pregnancy, (2) viable, single fetus, (3) age 18–38 years, (4) no previous pregnancy complications (e.g., preeclampsia, intrauterine fetal death, gestational diabetes, and preterm delivery), (5) no somatic or mental diseases (e.g., diabetes, kidney or cardiovascular diseases, and PCOS), and (6) no missed abortions or severe congenital anomalies.

Five women were excluded during the pregnancy: one had PCOS, three developed preeclampsia, and one experienced intrauterine fetal death at gestational week 35. The present study includes the remaining 119 healthy controls (Figure 1).

Healthy controls were examined according to the same protocol and at the same gestational weeks as PCOS women. One important difference was that healthy controls did not fast, did not take study medications, and were therefore not examined two hours after inclusion. They were also possibly examined at a later time of the day.

### 2.1. Ultrasound Measurements

All study participants were examined with Siemens ACUSON Antares™ (Siemens AG, Germany), Hitachi EUB-8500 (Hitachi Medisys, France), or Voluson 730 Expert (GE, Zipf, Austria) ultrasound devices. During Doppler ultrasound measurements, care was taken to avoid insonation of the embryo/fetus. Three experienced midwives specialized in obstetric ultrasonography and three experienced doctors (all working in the same unit) carried out all scans. The thermal (TI) and the mechanical indices (MI) were kept within the recommended thresholds, and the ISUOG guidelines for the safe use of Doppler ultrasound were followed [24].

The first visit was scheduled based on the last menstrual period. Crown rump length (CRL) was used to estimate gestational age at the time of the first examination. Estimated date of delivery and timing of the examination in week 24 were based on a second trimester routine ultrasound examination at approximately gestational week 18.

At the first three examinations for the PCOS women and the first two examinations for the healthy controls, the uterine artery pulsatility index (UtAPI) was measured with transvaginal ultrasound according to the following method: the uterine artery was identified at the point closest to the internal cervical os with the use of colour flow imaging. The sample gate was set at 2 mm and placed to cover the whole vessel, including the vessel walls. The angle was kept below 30 degrees. Three consecutive uniform waveforms were recorded, and the mean of the three was used. At the study visits at 19-20 weeks and 23-24 weeks, the UtAPI was measured with transabdominal ultrasound using the method described by Hernandez-Andrade et al. [25].

PI was measured three times on each side in order to reduce the effect of intraobserver variability, and the mean of all six measurements from the right and left uterine arteries was used in the final analyses.

### 2.2. Statistical Analysis

Baseline characteristics were analysed using the two sample *t*-test. Confidence intervals for mean PI were *t*-based while comparisons of mean PI between the control group and the PCOS group were done using a linear model with adjustment for gestational age. This was done because of the rapid change in UtAPI in early pregnancy and because gestational age at inclusion varied. We consider *p* values < 0.05 as statistically significant. We decided not to adjust for BMI or blood pressure, as these factors may be inherent components of PCOS. (We initially adjusted for maternal age and blood pressure, but this did not significantly change the result and we chose to keep the results as unadjusted as possible.) The statistical analysis was performed using R version 2.13.1 using the package lme4 and SPSS version 20 (IBM SPSS, Armonk, NY, USA).

## 3. Results

### 3.1. PCOS versus Controls

Women with PCOS and healthy control women had comparable sociometric parameters such as civil status, occupation, educational level, ethnicity, and parity (data not shown). The PCOS women were older and had higher BMI and blood pressure compared to the healthy controls (Table 1).

**Figure 2:**
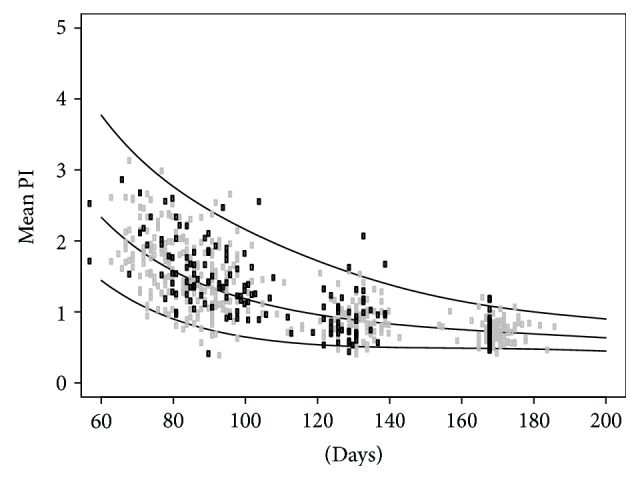
UtAPI percentile curve with 2.5, 50, and 97.5 percentiles. The lines are statistically calculated based on UtAPI measurements of the controls (gray rectangles) and PCOS women (black rectangles).

Women with PCOS and healthy controls were examined with UtAPI at the same point in time during first and second trimesters of pregnancy. We found no difference in mean UtAPI between the groups at any point in time that was investigated (Figure 2) (Table 2). Women with PCOS were randomly assigned to either metformin 2000 mg or placebo. We compared PCOS (metformin) and PCOS (placebo) groups separately to controls and found that there was no difference (Table 3).

### 3.2. PCOS

There was no difference in UtAPI between placebo and metformin groups 2 hours after intake of study medication (*p* = 0.34). UtAPI in the PCOS women was measured in the morning after an overnight fast and again 2 hours after intake of the first dose of study medication and a meal. After 2 hours, UtAPI was significantly increased (*p* = 0.018) in the PCOS group (both metformin and placebo groups). Analysing the PCOS (metformin) and PCOS (placebo) groups separately, mean UtAPI increased 0.13 in the placebo group (*p* = 0.18) and 0.18 in the metformin group (*p* = 0.05) (Table 4).

## 4. Discussion

We observed that women with PCOS and healthy controls had similar blood flow (UtAPI) in the uterine artery in the first and second trimesters and that food intake seems to impact UtAPI in pregnant women with PCOS.

Contrary to another comparable publication [15], we found no difference in UtAPI in the first and second trimesters of pregnancy between PCOS and healthy controls. Examinations were undertaken in a similar manner and by the same experienced personnel in both groups. One difference was that healthy controls did not fast before the 18-week examination and were scheduled for examination at random times during the day. PCOS women were examined in the morning and were fasting because of the PregMet study protocol. Before the study, we had no indication that fasting would affect blood flow [26, 27], and as far as we know, there are no other publications addressing this issue.

In a previous publication, we reported no long-term difference in UtAPI between placebo and metformin groups in the PregMet study (*N* = 270) [20]. When planning the present study, we read previous reports showing an increased uterine blood flow in PCOS women who received metformin, indicating that metformin could have a vessel-relaxing/dilating effect [18, 19, 28]. We found no reports on how quickly this could occur. Accordingly, in the present substudy, we aimed to investigate a possible immediate effect of metformin on the blood flow in women with PCOS. They received 500 mg metformin (a common start dose to avoid nausea), but the dose may have been too low to observe a cardiovascular effect. We found no difference in UtAPI two hours after intake of study medication between the placebo and metformin groups.

Surprisingly, we found higher mean UtAPI in both PCOS (metformin) and PCOS (placebo) groups at the examination done after 2 hours. Women were served a meal while waiting for the second examination. Studies in healthy pregnant women show no adverse effects of fasting on UtAPI [26, 27, 29]. These studies were conducted on women fasting for Ramadan and addressed long-term effects of fasting. Two groups (fasting and nonfasting) were compared at the beginning and the end of Ramadan, and one study was randomized. They found no difference in UtAPI between the two groups after one month of daytime fasting. None of the studies described when during the day, UtAPI was measured, that is, if the women were in a fasting state when the examination was done. We anticipate that they were not in a state of fast at the time of examination. This is because you are allowed to eat before sunrise and after sunset during Ramadan, and it is likely that women were examined during daytime and had ingested a meal before sunrise.

We were not able to find any studies on the effect of a meal or glucose load on UtAPI in pregnant women. We have previously demonstrated that high fasting blood glucose correlated inversely to UtAPI in the larger group of pregnant PCOS women from which the present subgroup was collected [20]. That is, the higher the fasting blood glucose, the lower the blood flow is. We can only speculate whether the expectedly higher blood glucose after a meal contributed to the higher UtAPI. In the nonpregnant state, food intake leads to vascular redistribution and shunting of blood to the gastrointestinal tract to promote digestion and absorption of nutrients. This leads to reduced blood flow and compensatory vasoconstriction in other areas of the body. This could also be the case in pregnant women and could be reflected in increased UtAPI as a measure of reduced blood flow. Other possible physiologic explanations could be diurnal variation in blood pressure and vasoconstriction induced by caffeine intake (coffee, tea, or coke).

PCOS is closely linked to metabolic syndrome and to increased prevalence of type 2 diabetes mellitus, gestational diabetes, and obesity [30]. Whole body metabolism including glucose metabolism seems to be important in the pathogenesis of PCOS. Exact mechanisms have yet to be clarified.

## 5. Conclusion

Blood flow in the uterine artery does not seem to differ between women with PCOS and healthy controls. Metformin does not have any immediate effect on UtAPI, but we observed that UtAPI was significantly higher two hours after a meal in pregnant women with PCOS. Standardization of food intake should be considered in future studies measuring UtAPI.

## Figures and Tables

**Table 1:** Baseline characteristics of the study participants.

	PCOS	Controls	*p* values
Age (years)	29.9 (4.6)	27.9 (4.2)	0.01
BMI (kg/m^2^)	29.0 (7.6)	24.1 (4.2)	<0.001
Systolic BP (mmHg)	120 (10.6)	114 (11.6)	0.001
Diastolic BP (mmHg)	78 (9.3)	68 (10.0)	<0.001

Values are given as means and standard deviation (SD).

**Table 2:** Number and time of UtAPI measurements.

PCOS women	Healthy controls
(1a) Inclusion (1st trimester)	(1) Inclusion
(1b) Inclusion + 2 hours	
(2) Inclusion + 2 weeks	(2) Inclusion + 2 weeks
(3) Week 18	(3) Week 18
(4) Week 24	(4) Week 24

**Table 3:** Mean UtAPI values and number of examinations at each given study point. In the analysis, we adjusted for gestational age.

	Controls	PCOS	PCOS	PCOS	*p* value
	(*n*)	All (*n*)	Placebo (*n*)	Metformin (*n*)	
Mean UtAPI inclusion	1.79 (118) CI (1.70–1.87)	1.73 (46) CI (1.58–1.88)	1.80 (24) CI (1.58–2.03)	1.66 (22) CI (1.44–1.88)	PCOS all *p* = 0.34 PCOS placebo *p* = 0.18 PCOS metformin *p* = 0.93
Mean UtAPI inclusion + 2 weeks	1.41 (114) CI (1.33–1.49)	1.37 (43) CI (1.25–1.49)	1.41 (23) CI (1.23–1.60)	1.31 (20) CI (1.13–1.49)	PCOS all *p* = 0.77 PCOS placebo *p* = 0.46 PCOS metformin *p* = 0.62
Mean UtAPI week 18	0.89 (109) CI (0.85–0.94)	0.93 (45) CI (0.83–1.03)	1.00 (24) CI (0.84–1.17)	0.83 (21) CI (0.73–0.93)	PCOS all *p* = 0.47 PCOS placebo *p* = 0.08 PCOS metformin *p* = 0.28
Mean UtAPI week 24	0.73 (108) CI (0.71–0.76)	0.75 (33) CI (0.68–0.82)	0.81 (17) CI (0.69–0.92)	0.70 (16) CI (0.62–0.77)	PCOS all *p* = 0.68 PCOS placebo *p* = 0.12 PCOS metformin *p* = 0.28

**Table 4:** At inclusion (first trimester) UtAPI was measured after an overnight fasting period, then 2 hours later after ingesting the study medication and a meal.

	Mean UtAPI PCOS (SD)	Mean UtAPI PCOS (SD)	*p* value
	Fasting	After meal	
PCOS all	1.57 (0.50)	1.73 (0.51)	*p* = 0.02
PCOS placebo	1.66 (0.57)	1.80 (0.54)	*p* = 0.18
PCOS metformin	1.47 (0.42)	1.66 (0.50)	*p* = 0.05
